# The Hidden Threat: Chronic Urinary Retention and Thromboembolism in Underlying May–Thurner Syndrome

**DOI:** 10.1155/crvm/1579198

**Published:** 2025-03-06

**Authors:** Tanner M. Dunn, Clint A. Hayes

**Affiliations:** ^1^Texas College of Osteopathic Medicine, University of North Texas Health Science Center, Fort Worth, Texas, USA; ^2^Vascular Surgery, Vein Center of North Texas, Sherman, Texas, USA

## Abstract

May–Thurner syndrome (MTS) is characterized by the formation of an intravenous scar or venous “spur” resulting from chronic pulsatile compression of the left common iliac vein (LCIV) by the right common iliac artery (RCIA) against the fourth or fifth lumbar vertebral body. This pulsatile compression creates a flow-limiting stenosis of the LCIV, leading to increased intravenous pressure in the veins draining the left lower extremity (LLE). Consequently, this elevated venous pressure manifests as a spectrum of chronic symptoms including unilateral LLE edema, pain, tenderness, warmth, skin inflammation, and discoloration, along with pelvic symptoms such as sensation of fullness and dyspareunia. Furthermore, MTS significantly elevates the risk of venous thromboembolism characterized by LCIV deep vein thrombosis (DVT) and potentially fatal pulmonary embolism (PE). Treatment options for MTS range from anticoagulant therapy to, in severe cases, operative intervention. Herein, we present a case of a 79-year-old male with MTS who experienced a near-fatal DVT that led to PE, compounded by a chronically distended urinary bladder, necessitating immediate operative removal of the PE.

## 1. Introduction

In 1957, May and Thurner observed and defined the phenomenon known today as May–Thurner syndrome (MTS) or iliocaval compression syndrome. Typically, as the aorta descends, it bifurcates into the right and left common iliac artery, while the inferior vena cava (IVC) is formed by the convergence of the right common iliac vein (RCIV) and left common iliac vein (LCIV). This anatomical configuration is noteworthy because it renders the delicate LCIV susceptible to compression by the high-pressure, pulsatile right common iliac artery (RCIA). The venous “spur” formation, as described by May and Thurner, is a direct result of repeated, chronic insult to the vascular tissue of the LCIV by the RCIA's rhythmic compression against the lumbar vertebrae [1]. While most patients with MTS are asymptomatic, when symptoms do develop, patients may present with unilateral left lower extremity (LLE) edema; pain; skin discoloration; venous claudication; deep vein thrombosis (DVT); and in rare cases, PE [2]. We report the case of a 79-year-old male with MTS, where a previously undiagnosed and chronically distended urinary bladder further complicated his condition, leading to the emergent need for operative removal of a 9-cm PE and implementation of lifelong anticoagulation.

## 2. Case Presentation

A 79-year-old white male presented to the emergency department in acute distress after awakening with severe shortness of breath. He denied chest pain, fever, chills, paroxysmal nocturnal dyspnea, and leg pain. He reported traveling more than 8 h by car twice in the previous week. His past medical history included hypertension, hyperlipidemia, coronary artery disease, and benign prostatic hyperplasia (BPH), previously treated with transurethral resection of the prostate (TURP). He had no history of asthma or COPD, and his only medication was simvastatin. Physical examination revealed a well-developed, well-nourished individual in acute distress who was alert, anxious, and ill-appearing with hypoxia (oxygen saturation (SpO_2_) as low as 80%), hypotension, and unilateral LLE tenderness and edema. He had no cough, hemoptysis, peripheral edema, diaphoresis, or palpitations. Cardiovascular examination revealed orthopnea and tachycardia without murmurs. Respiratory examination demonstrated tachypnea, diminished breath sounds bilaterally in the lower posterior lung fields, moderate retractions, and otherwise clear lung sounds with no chest wall tenderness.

**Figure 1:**
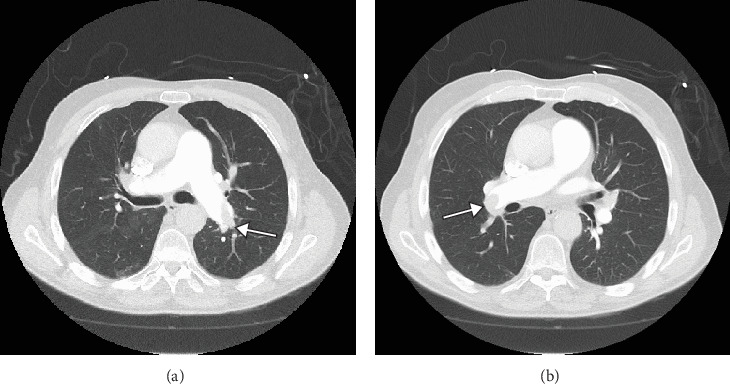
Thrombus in the left (a) and right (b) main pulmonary arteries.

Echocardiogram indicated a dilated right ventricle. Troponin levels were elevated and trended over time, starting at 0.23 ng/mL on presentation, increasing to 0.43 ng/mL after 6 h, and rising further to 0.56 ng/mL 3 h later. Lower extremity duplex examination revealed occlusive DVT in the left common femoral vein and left posterior tibial vein. A CT angiogram showed extensive central pulmonary emboli measuring 9 cm in the right and left pulmonary arteries, necessitating immediate evacuation (Figure 1). The patient's Pulmonary Embolism Severity Index (PESI) score of 129 classified him as Class V, indicating a significantly elevated risk of mortality and complications, warranting emergent intervention. Due to his unstable presentation, including shortness of breath, hypoxia, and hypotension, he was promptly taken to the catheterization lab for emergent thrombolysis and clot evacuation. A low-dose thrombolytic infusion was administered with alteplase delivered via catheter at a rate of 0.5–1 mg/h. The infusion rate and duration were adjusted based on angiographic findings and hemodynamic response, ensuring effective clot dissolution while minimizing systemic bleeding risks. Following the procedure, the patient experienced immediate symptomatic relief and was discharged home on lifelong apixaban 5 mg twice daily to prevent recurrence.

**Figure 2:**
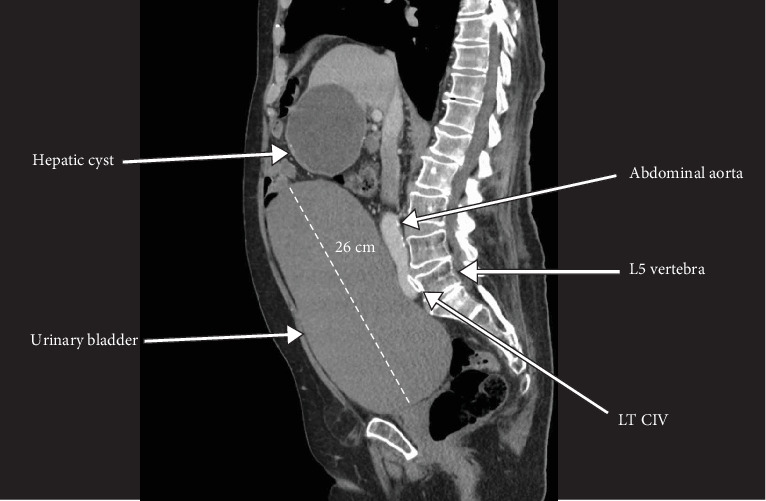
Sagittal section showing markedly distended urinary bladder compressing the right common iliac artery against the left common iliac vein. LT CIV, left common iliac vein.

**Figure 3:**
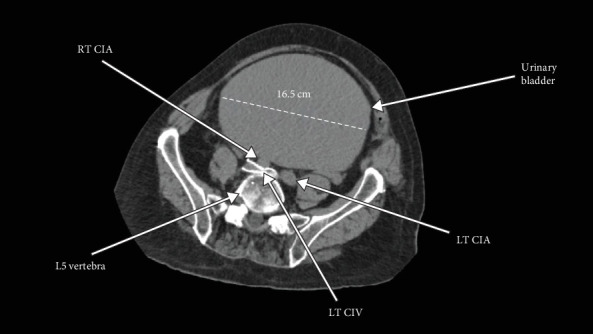
Transverse section showing markedly distended urinary bladder compressing the right common iliac artery against the left common iliac vein. RT CIA, right common iliac artery; LT CIA, left common iliac artery; LT CIV, left common iliac vein.

**Figure 4:**
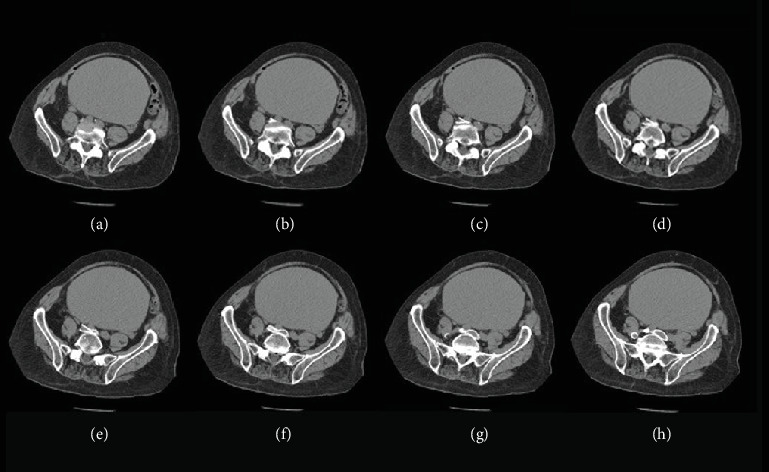
(a–h) Transverse series showing the urinary bladder compressing the right iliac artery against the left iliac vein at the level of the fifth lumbar vertebra.

**Figure 5:**
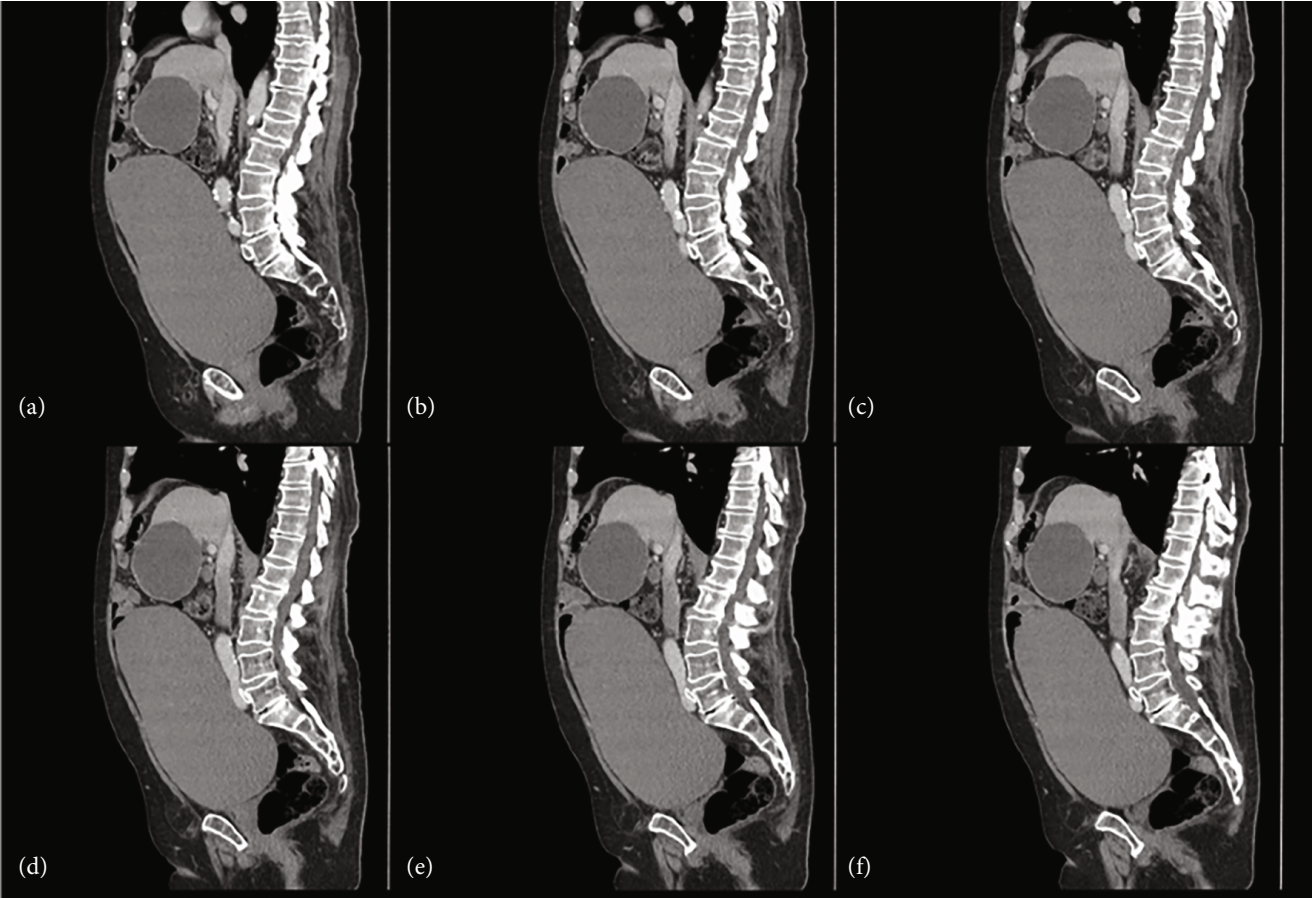
(a–f) Sagittal series showing the urinary bladder compressing the right iliac artery against the left iliac vein at the level of the fifth lumbar vertebra.

Given the presenting DVT/PE, a CT abdomen with contrast was performed weeks after the initial embolectomy, revealing a markedly distended urinary bladder measuring approximately 26 cm long, 16.5 cm anteroposteriorly, and 16.5 cm wide (Figure 2). The bladder size was suggestive of a chronic urinary bladder distention (Figure 3) producing significant compression upon laterally and posteriorly displaced arterial and venous vessels with the posteriorly displaced RCIA producing severe compression and narrowing of the LCIV against the fifth lumbar vertebrae as it coursed across the midline (Figures 4 and 5). The abdominal aorta showed scattered atherosclerotic calcifications without any evidence of aneurysm, and a large, unrelated hepatic cyst was observed.

**Figure 6:**
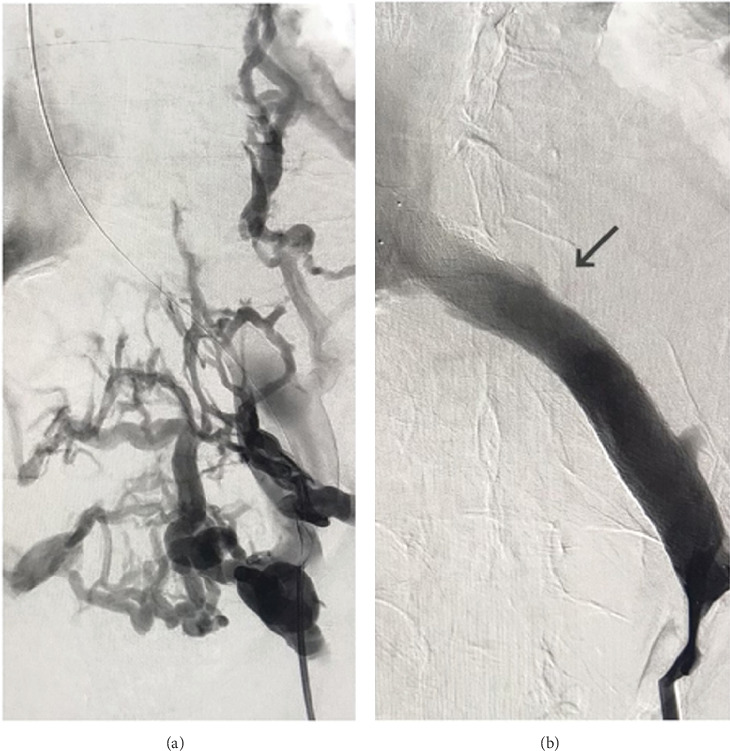
(a) Venogram with digital subtraction angiography depicting pelvic collaterals which were visualized with contrast. (b) Venogram with digital subtraction angiography depicting high-grade stenosis with complete occlusion of the left common iliac vein.

Venogram with digital subtraction angiography (DSA) and percutaneous transluminal angioplasty (PTA) with stent placement were used to further identify and treat the patient's MTS. Venography demonstrated a high-grade stenosis with 100% occlusion of the LCIV (Figure 6(b)). Pelvic collaterals were visualized with contrast (Figure 6(a)). Due to the chronicity of the patient's MTS, extensive pelvic venous collateralization had developed, rerouting flow from the LCIV to the left internal iliac vein. This flow entered the overdeveloped pelvic collaterals and moved into the right internal iliac vein. Eventually, collateral flow progressed into the RCIV and ultimately the IVC. PTA of the LCIV stenosis with stent placement successfully addressed the chronic occlusion, resulting in immediate symptom relief. To further manage the patient's chronic urinary retention, he was instructed to perform intermittent self-catheterization twice daily, reducing bladder distention and minimizing venous compression while avoiding long-term indwelling catheter use.

At follow-up visits conducted at 1 week, 1 month, and 6 months postprocedure, the patient demonstrated sustained symptom resolution without any recurrence of LLE edema, pain, or tenderness. The patient adhered to the prescribed anticoagulation therapy with no evidence of stent migration, restenosis, or thrombotic complications observed.

## 3. Discussion

MTS, or iliac vein compression syndrome, is a widely underdiagnosed vascular condition that can lead to the formation of a DVT and fatal PE. Classic MTS is characterized by the compression of the LCIV by the RCIA against the fourth or fifth lumbar vertebral body. Chronic pulsations of the LCIV against the lumbar vertebrae by the RCIA generate an intraluminal scar known as a venous “spur.” In 1908, McMurrich was the first to describe this phenomenon, and in 1957, May and Thurner were the first to define it [1, 3].

Acute PE is a glaring complication of long-standing DVT associated with MTS. Each year, around 900,000 Americans are estimated to be affected by venous thromboembolism, a serious medical problem involving DVT and PE [4]. Of this, an estimated 300,000 patients are hospitalized per year for PE with 10%–30% of individuals dying within 1 month of diagnosis [5, 6]. The PE experienced by our patient represents a rare complication of MTS with studies showing that the incidence of MTS-related DVT is estimated to be 2%–3%. Similar studies point out that a diameter of more than 70% stenosis of the LCIV is present in 30%–40% of the general population, postulating that MTS is more common than previously thought [7–9].

Studies show that women are twice as likely to have MTS and six times more likely to develop PE [10]. This is especially true of those with a prior history of pregnancy or oral contraceptive use, suggesting that screening may be warranted when left-sided DVT presents during oral contraceptive use or the peripartum period [11]. Other risk factors for venous thrombosis include surgery, injury, or conditions predisposing to a hypercoagulable state [12].

Few studies have reported MTS exacerbated by chronic urinary retention leading to DVT and near-fatal PE in men. Urinary retention may develop in men and women due to bladder outlet obstruction or detrusor contractility abnormalities [13]. Chronic urinary retention is linked to a higher prevalence in men. Compared to women, men are 13 times more likely to suffer from urinary retention [14, 15]. This is due to the exclusivity and prevalence of BPH in the male population, with recent results suggesting that 1 in 4 men will suffer from BPH over their lifetime [16].

To prevent death in acute DVT/PE, prompt diagnosis must be made when symptoms appear. Studies indicate that DVTs are predominantly left-sided in 55.9% of cases. Symptoms manifest as acute or chronic unilateral LLE edema, pain, tenderness, warmth, skin discoloration, enlarged or swollen superficial veins, and venous claudication [2].

Both CT and magnetic resonance venography are the largely preferred modalities for diagnosing MTS with the ability to rule out bone or vascular anomalies and pelvic pathologies [17, 18]. However, venography with intravascular ultrasound (IVUS) remains the gold standard for diagnosing and guiding therapy for MTS [19, 20]. A combination of PTA and a catheter-directed thrombolysis, followed by iliac vein stenting with subsequent anticoagulation, is the most reported method of treatment in the literature at this time [21].

## 4. Conclusion

Diagnosis of MTS requires a heightened index of clinic suspicion when observing patients presenting with signs of unilateral left leg swelling and pain, those with long-standing pelvic symptoms (especially in women), or those with unexplained or recurrent DVTs. Risk factors such as prior pregnancies or oral contraceptive use in women, and a history of BPH in men, should be considered when evaluating for MTS. Early recognition is important for preventing potentially fatal complications such as PE. This case underscores the importance of considering variable etiologies of MTS, such as chronic urinary retention, in the differential diagnosis and early treatment of thromboembolic events.

## Data Availability

Data sharing is not applicable to this article as no new data were created or analyzed in this study.
